# Multistage Centrifugal Pump Fault Diagnosis Using Informative Ratio Principal Component Analysis

**DOI:** 10.3390/s22010179

**Published:** 2021-12-28

**Authors:** Zahoor Ahmad, Tuan-Khai Nguyen, Sajjad Ahmad, Cong Dai Nguyen, Jong-Myon Kim

**Affiliations:** 1 Department of Electrical, Electronics and Computer Engineering, University of Ulsan, Ulsan 44610, Korea; zahooruou@mail.ulsan.ac.kr (Z.A.); khaint@mail.ulsan.ac.kr (T.-K.N.); sajjad907@mail.ulsan.ac.kr (S.A.); dainc@mail.ulsan.ac.kr (C.D.N.); 2 Predictive Diagnosis Technology Cooperation, Ulsan 44610, Korea

**Keywords:** fault diagnosis, multistage centrifugal pump, principal component analysis

## Abstract

This study proposes a fault diagnosis method (FD) for multistage centrifugal pumps (MCP) using informative ratio principal component analysis (Ir-PCA). To overcome the interference and background noise in the vibration signatures (VS) of the centrifugal pump, the fault diagnosis method selects the fault-specific frequency band (FSFB) in the first step. Statistical features in time, frequency, and wavelet domains were extracted from the fault-specific frequency band. In the second step, all of the extracted features were combined into a single feature vector called a multi-domain feature pool (MDFP). The multi-domain feature pool results in a larger dimension; furthermore, not all of the features are best for representing the centrifugal pump condition and can affect the condition classification accuracy of the classifier. To obtain discriminant features with low dimensions, this paper introduces a novel informative ratio principal component analysis in the third step. The technique first assesses the feature informativeness towards the fault by calculating the informative ratio between the feature within the class scatteredness and between-class distance. To obtain a discriminant set of features with reduced dimensions, principal component analysis was applied to the features with a high informative ratio. The combination of informative ratio-based feature assessment and principal component analysis forms the novel informative ratio principal component analysis. The new set of discriminant features obtained from the novel technique are then provided to the K-nearest neighbor (K-NN) condition classifier for multistage centrifugal pump condition classification. The proposed method outperformed existing state-of-the-art methods in terms of fault classification accuracy.

## 1. Introduction

The multistage centrifugal pump (MCP) converts electrical energy to mechanical energy for industrial processes [1]. MCP is the type of centrifugal pump in which multiple impellers are fitted in series and the fluid flows through the series of impellers. A survey conducted on 437 defective MCPs revealed that the industry went through a maintenance downtime of 6128 h because of the lack of intelligent FD, resulting in a cost of USD 50 million [2]. Defects in the MCP can be categorized into soft defects and hard defects. Hard defects occur abruptly and cause the MCP to stop unexpectedly. Hard defects are physically identifiable and can be addressed by inexpensive and simple analysis; however, soft defects are dangerous as they slowly affect the MCP performance. Thus, soft defects need to be identified quickly using intelligent FD [3]. Mechanical seal (MS)-related defects are responsible for 34% of the MCP soft defects. A defective MS results in MCP soft defects such as shaft wear, flushing of fluid, and fretting, etc. Furthermore, a defective impeller can cause hydraulic soft defects and mechanical soft defects [4,5]. To reduce downtime and cost for MCP maintenance, this paper considers the early fault diagnosis of soft defects because of a MS hole (MSH), MS scratch (MSS), and impeller defect (ID).

A change in the stiffness of the mechanical structure because of a mechanical defect produces an impulse in the VS. Therefore, VS can be used for the condition monitoring of MCP [6]. The impulses in the VS because of a mechanical defect occur at a specific frequency in the Fourier spectrum (FS). However, because of the low energy of the fault impulses, they are often obscured by interference and background noises [7]. Andrei et al. [8] discriminated the bearing fault harmonics from interference noises using the fault-oriented window series of a Gaussian mixture model. However, techniques based on narrowband demodulation are unable to discriminate between interference noise and fault impulses [9,10]. Furthermore, the VS under a defect changes its statistical characteristic over time indicating they are highly complex and nonstationary. Fourier transforms are ideal for stationary signals. To address these concerns, Viet et al. [11] proposed a blind source separation (BSS) technique-based acoustic signal denoising. However, the BSS requires a baseline signal for noise reduction in the subsequent signal. For complex VS, time-frequency domain (TFD) transforms have significant advantages. The TFD wavelet transform (WT) is sensitive to non-stationary defect impulses [12,13,14]. Rapur and Tiwari [15] preprocessed the VS of the MCP using WT and extracted statistical features (SF) for MCP fault diagnosis. For WT, an optimal mother wavelet selection for VS preprocessing is very important, otherwise the WT will suffer from an oscillation effect. Empirical mode decomposition (EMD) [16,17,18], an adaptive signal decomposition technique, can overcome the shortcomings of WT. Unfortunately, EMD has mode mixing and it suffers from extreme interpolation. These drawbacks of EMD make the WT more attractive [19]. To address the above concerns, rather than focusing on a narrow optimal frequency band, this paper first calculates the modes of vibration for MCP defects. To overcome the interference macrostructural vibration noise, these MCP defect modes of vibration are filtered from the MCP vibration spectrum. The filtered mode of vibration forms the FSFB, which is then used for discriminant SF extraction.

After VS preprocessing, feature extraction and feature preprocessing are important steps in intelligent FD [20,21,22]. SF can be extracted from the VS in time, frequency, and TFD [23]. The power of deep learning techniques can be utilized for fault-related discriminant feature extraction and classification [24,25]. Juan et al. [24] proposed a data-driven fault diagnosis strategy for bearing fault diagnosis. The proposed method extracts SF’s from the raw vibration signal in the time domain, frequency domain, and TFD. To obtain the discriminant set of features with reduced dimensions for the identification of bearing working conditions, a novel deep feature learning technique is proposed. However, the interaction of complex fluid and mechanical components inside the pump and the stiffness change in the mechanical component of the CP change the nature of the VS obtained from the CP under soft defect conditions from the vibration signal of the bearing. Thus, SF’s extracted from the raw VS of the CP in multiple domains result in noisy features. Furthermore, they are not capable to represent the fault-related information of the CP. Moreover, the time domain SF’s extracted from the raw vibration signal are either not sensitive to incipient defects or are not appropriate for severe defects [26]. In the case of the frequency domain, SF’s extracted from the frequency spectrum of the raw vibration signal may be noisy because the fault-related frequencies of the CP often occur at lower frequencies, and therefore, it can be overwhelmed by microstructural vibration noise. To address these concerns, a new technique is introduced for CP VS pre-processing which is applied to the raw VS before feature extraction. The technique first calculates the modes of vibration for MCP defects. These MCP defect modes of vibration are filtered from the MCP vibration spectrum. The filtered mode of vibration forms the FSFB, which is then used for discriminant SF’s extraction in time, frequency, and TFD. Yet the study presented in [24] is interesting and can be used for CP fault diagnosis after VS pre-processing. The SF’s extracted from time, frequency, and TFD are combined into a single feature vector called MDFP. The MDFP results in a larger dimension. Furthermore, not all features are best for representing centrifugal pump conditions and they can affect the condition classification accuracy of the classifier. To address this concern, feature preprocessing for discriminant feature extraction is of primary importance [27,28,29,30,31,32,33,34,35]. Several feature dimensionality reduction and discriminancy evaluation techniques have been proposed [36,37,38]. Among them, principal component analysis (PCA) and linear discriminant analysis (LDA) are most common. LDA preprocesses the feature and results into discriminant feature space by reducing within class feature sparseness and increasing the feature distance between classes [39]. Several variants of LDA are proposed such as Pearson LDA [40] and trace ratio LDA (Tr-LDA) [41]. However, the penalty graph representation for between classes distance affects the discriminant of the feature space. In contrast, the PCA constructs low dimensional feature space by considering the variance. Sakthivel et al. [42] evaluated supervised and unsupervised dimensionality reduction techniques for MCP fault classification. The evaluation showed that PCA outperformed all feature preprocessing methods for MCP fault classification. Unfortunately, PCA does not consider between class feature distance or within class feature sparseness. For this reason, this paper proposes a novel Ir-PCA. The novel technique first assesses the feature informativeness towards the fault by calculating the informative ratio between the feature within the class scatteredness and between class distance. To obtain a discriminant set of features with reduced dimensions, PCA is applied to the features with a high informative ratio. The combination of informative ratio-based features assessment and PCA forms the novel Ir-PCA.

The overall contribution of this work can be summarized as follows:

1. To overcome the macrostructural interference noises, this paper first calculates the vibration modes for MCP defects. These MCP defect modes of vibration are filtered from the MCP vibration spectrum. The filtered mode of vibration forms the FSFB, which is used for discriminant SF extraction in time, frequency, and TFD. All of these SF were combined into a single feature victor called MDFP.
2. Ir-PCA was proposed for discriminant feature extraction for MCP fault diagnosis. To the best of our knowledge, Ir-PCA has not been reported. Ir-PCA first assesses the feature informativeness towards the fault by calculating the informative ratio of the features. To obtain a discriminant set of features with reduced dimensions, PCA was applied to the features with a high informative ratio.
3. The MCP vibration signal obtained from a real-world industrial test rig was used for the evaluation of the proposed method.

The paper is organized into the following sections. Section 2 presents the technical review. The pump experimental test rig is presented in Section 3. Section 4 explains the proposed method. Section 5 presents the performance evaluation of the proposed method. Section 6 presents the conclusion of this study.

## 2. Technical Review

### 2.1. Review of Principle Component Analysis

PCA is one of the most popular methods when dimensionality reduction is concerned. Two criteria are essential in a dimensionality reduction method: the ability to compress the data and simultaneously maximize the coverage of data variances. PCA’s approach to these tasks is constructed around the finding of the reduced dimensionality’s linear basis.

Given an *Nx1* vector *x*_1_, *x*_2_, …, *x_n_*, the general scheme of PCA initiates with the removal of the mean value $\bar{x}$ from each feature, which is used for computing the covariance matrix $C_{\mathrm{cov}}$ that represents the data distribution. $C_{\mathrm{cov}}$ eigenvalues and eigenvectors can be achieved using Equations (1) and (2).

(1) $$C_{EV}=\lambda_{i}>\lambda_{i+1}>...\lambda_{N}$$

(2) $$C_{EVec}=\mu_{i}>\mu_{i+1}>...\mu_{N}$$

Thus, (*x_i_* − $\bar{x}$) can be rewritten as Equation (3):

(3) $$x_{i}-\bar{x}=a_{1}\mu_{1}+a_{2}\mu_{2}+...+a_{N}\mu_{N}=\sum_{i=1}^{N}l$$

where *a*_1_, *a*_2_, …, *a_n_* are scalars. Finally, only the corresponding terms of the *K* largest eigenvalues are retained, as shown in Equation (4):

(4) $$x_{i}-\bar{x}=\sum_{i=1}^{N}a_{i}-\mu_{i}\;\mathrm{where}\;K\ll N$$

### 2.2. Review of the Wavelet Packet Transform (WPT)

**Figure 1:**
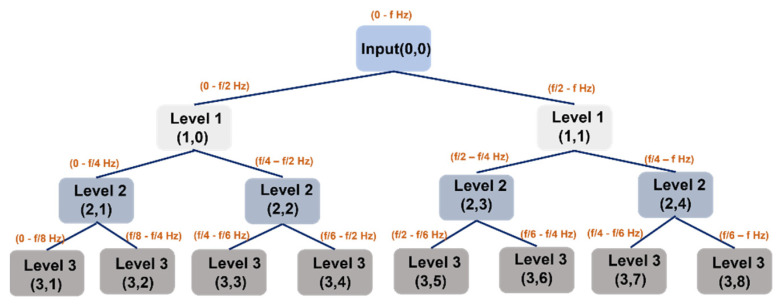
WPT decomposition tree up to 3 levels.

Being an extension of the original wavelet transform (WT), WPT also presents a solution to independent frequency band analysis. Instead of dividing the signal into *k* levels, such as WT, WPT breaks the signal using low- and high-pass filters while creating 2*^k^* nodes at each level. As a result, WPT can exceed WT in terms of resolution, thus obtaining a more comprehensive analysis of time–frequency across the signal spectrum. Its coefficients can be computed as follows:

(5) $$c_{k+1}^{j}(n)=c_{k}^{j}\times h(-2n),\;\;\;0<j<2^{k}-1$$

(6) $$d_{k+1}^{2j+1}(n)=d_{j}^{k}(n)\times g(-2n),\;\;0<j<2^{k}-1$$

where *h* and *g* are the low- and high-pass filters, respectively, *k* is the levels (scale parameter), and 2*j,* 2*j +* 1 are the nodes (frequency parameter) in Equations (5) and (6). Figure 1 shows the algorithmic description of WPT decomposition for three levels. A detailed description of WPT can be found in [43].

## 3. Pump Experimental Test Rig

**Figure 2:**
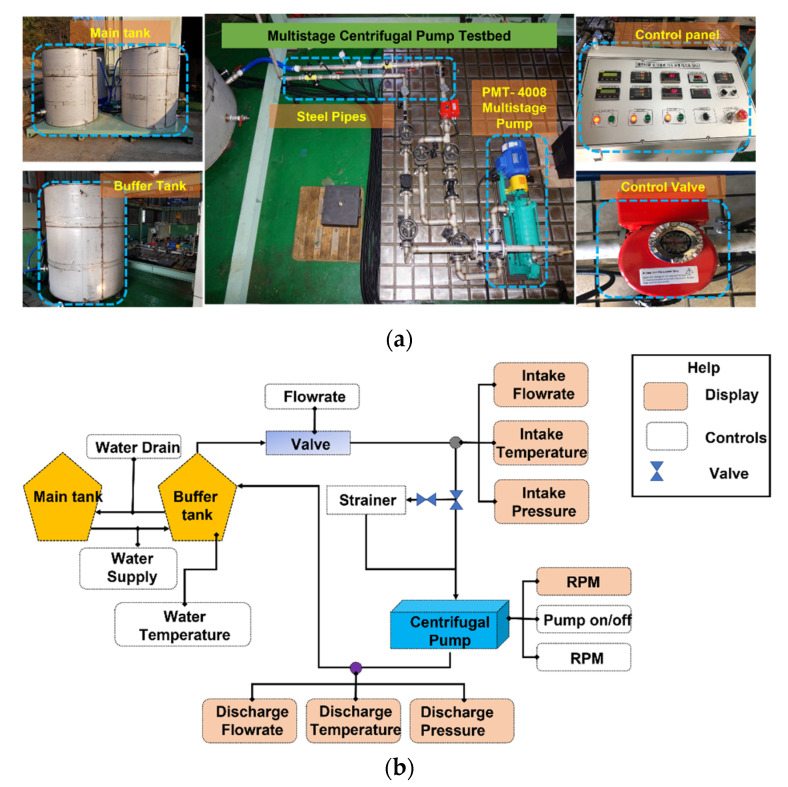
Pump test rig: (**a**) photograph and (**b**) schematics.

The MCP test rig setup picture and schematics are shown in Figure 2a,b, respectively. The pump test rig consists of an MCP PMT-4008 (Hanil, Gwangju, Korea) driven by a 5.5 kW motor. A user-friendly control panel was established for controlling the pump speed, power, and flow rate. After turning on the electric power, the MCP starts pumping water from the main tank to the buffer tank through steel pipes. The VS were recorded from the MCP at a speed of 1733 rpm using accelerometers.

**Figure 3:**
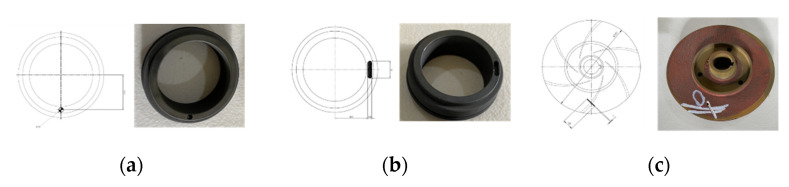
MCP defects: (**a**) MSH, (**b**) MSS, and (**c**) IF.

A total of four accelerometers were used for recording the VS of the MCP. Two of the accelerometers were attached to the pump casing, one accelerometer near the impeller in the axial direction, and one near the mechanical seal in the axial direction. A 300 Seconds (s) long VS was recorded from the MCP in the normal condition (NC). After collecting the MCP data in NC, the rotating part of the MS was replaced by a defective MS, as shown in Figure 3a. An MSH with a diameter of 2.8 mm and depth of 2.8 mm in the MS was created using an electrical drill, and the VS from the MCP were recorded for 300 s. Similarly, an MSS was created in the rotating part of the MS with a 38 mm inner diameter, as presented in Figure 3b, and the VS from the MCP were collected for 300 s. An ID, as shown in Figure 3c, was created by removing a metal piece from the impeller using an electric device with a length of 18 mm and a depth of 2.8 mm; the VS from the MCP were collected for 300 s.

**Figure 4:**
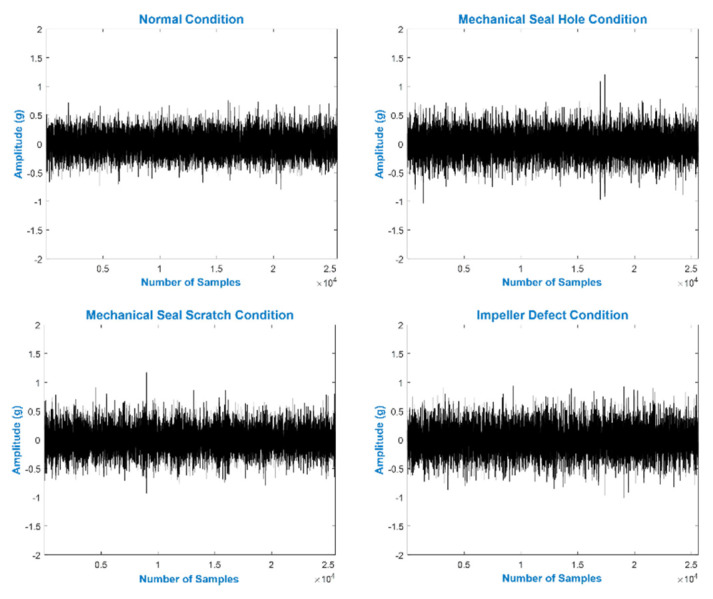
VS of the MCP under NC and defective conditions.

A total of 1200 s of VS were collected from the MCP. All of the VS were digitized using a NI-9234 DAQ. Figure 4 shows the VS obtained from the MCP in NC and a defective condition.

## 4. Proposed Fault Diagnosis Method

**Figure 5:**
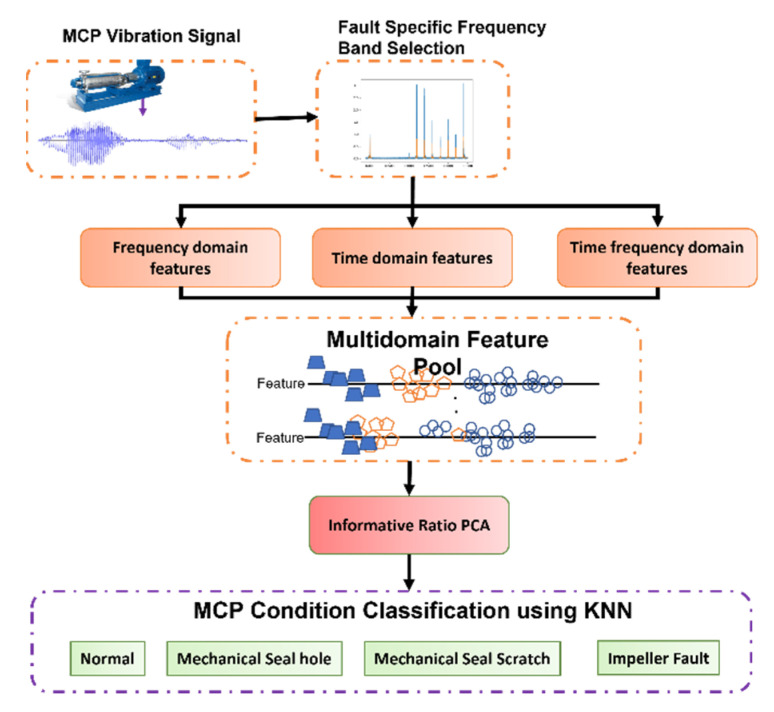
Proposed method for MCP condition monitoring.

The proposed method starts with selecting FSFB and ends with MCP health state classification. Figure 5 shows the flow diagram of the proposed method. Following are the steps involved in the proposed method.

### 4.1. Step 1: Fault Specific Frequency Band Selection

A fault can be identified from the shock produced by the change in stiffness around the mechanical structure when the fault occurs. Although the observation in the frequency spectrum can theoretically allow us to speculate these shocks at the fault-specific frequencies (FSF), it is more sophisticated in the case of the CP. The increased complexity is because of the interactions between the fluid and mechanical components, which can cause a hydraulic defect from the original mechanical defects. As a result, discriminant features might not be achieved with the sole focus on FSF. Furthermore, raw VS can be affected by the macrostructural vibration, thus altering the SF quality. These issues are addressed in this step by the identification and selection of FSFB.

**Figure 6:**
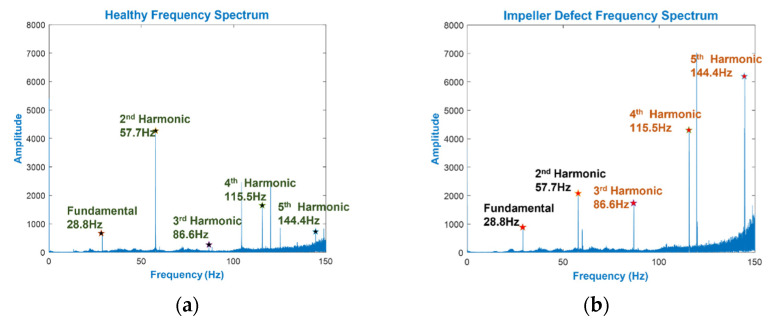
MCP spectrum of (**a**) NC, and (**b**) ID.

Three main types of frequency are of interest in the CP: generated, excitation, and electronics. The harmonics from the first two types are considered valuable features concerning this study’s objective. The identification of these frequencies is feasible because of the system parameters (i.e., rotating speed, CP geometry, etc.). When the defect is located on the impeller, the generated frequencies are the source. The impeller imbalance caused by this defect can be observed in the VS [44], which is described at an FSF by the following equation:

(7) $$F_{ID}=n\cdot Z$$

where *n* is the frequency harmonics and *Z* is the MCP’s operating speed (Hz) in Equation (7). The FS difference of an MCP in the normal condition and ID condition are demonstrated in Figure 6. As *F_ID_*’s calculation was taken to the 5th harmonic, the increase in the amplitude of the last three was attributed to the ID. Moreover, in the case of ID, spikes were observed across the FS, which can be explained by the interaction between the defective impeller and fluid.

In the MCP spectrum, an MS defect is associated with the excitation frequency, which reflects the CP’s amplified vibration as a single frequency harmonic [5,45]. The circular ring vibration theory was used to explain the calculation of the excitation frequency, which starts with the calculation of the deformation potential energy (PE) and vibration kinetic energy (KE):

(8) $$E_{PE}=\left(\frac{AEu_{r}{}^{2}}{2r^{2}}\times 2\pi r\right)$$

(9) $$E_{KE}=\left(\frac{\rho A}{2}\right)\left({\left(u_{r}{}^{\prime }\right)}^{2}\times 2\pi r\right)$$

where *A* is the cross-sectional area of the ring, *u_r_* is the radial displacement, *r* is the centerline radius of the ring, and *E* is the elasticity modulus. *u/r* represents the ring’s unit elongation in Equations (8) and (9). According to the energy conservation method:

(10) $$\frac{d}{dt}((E_{PE}+E_{KE})=0$$

By solving Equation (10), the equation of motion is achieved as:

(11) $$({u^{\prime \prime }}_{r})+(\omega_{{}^{\circ}}^{2})u_{r}=0$$

where $\omega_{{}^{\circ}}^{}$ is the angular frequency. Equation (11) can then be solved, which results in the ring fundamental frequency as Equation (12):

(12) $$f_{rf}=\left(\frac{1}{2\pi r}\right)\sqrt{\frac{E}{\rho }}$$

**Figure 7:**
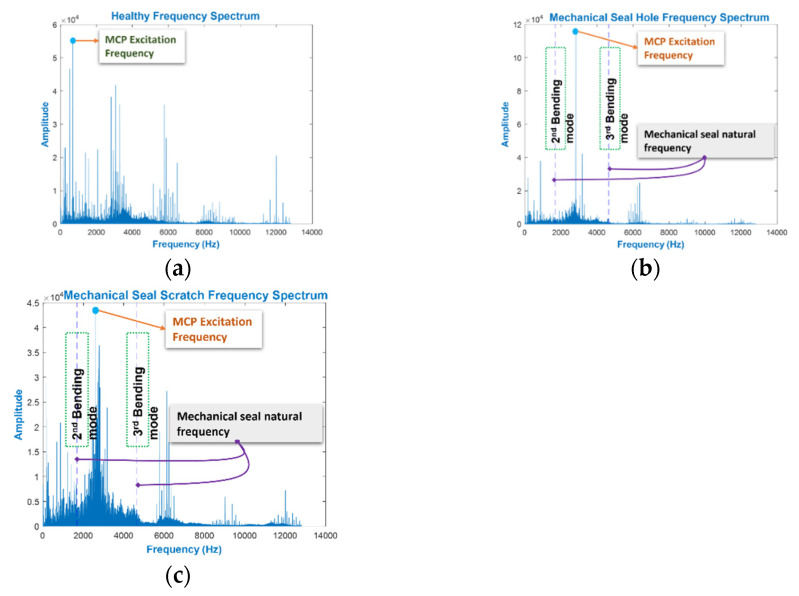
MCP spectrum of (**a**) NC, (**b**) MSH, and (**c**) MSS.

Appropriate modes are present because the vibration is nonrandom. For a MS, the expressions of in-plane and out-of-plane modes are:

(13) $$f_{inplane}=\left(\frac{2n\left(n^{2}-1\right)}{\pi }\right)\left(\frac{h}{d^{2}}\right)\sqrt{\frac{E}{\rho \left(12n^{2}\right)+\left(\frac{2th^{3}\left(1+\nu \right)}{c}\right)}}$$

(14) $$f_{inplane}=\left(\frac{2n\left(n^{2}-1\right)}{\pi }\right)\left(\frac{h}{d^{2}}\right)\sqrt{\frac{E}{\rho \left(12n^{2}\right)+\left(\frac{2th^{3}\left(1+\nu \right)}{c}\right)}}$$

where *h* is the cross-sectional height, *t* is the ring thickness, *d* is the diameter, *v* is Poisson’s ratio, *c* is the torsion constant, and *n* is the vibration mode. Generally, high frequencies occur where the fundamental and in-plane vibrations occur. Nevertheless, lower frequencies were witnessed in the case of out-of-plane bending modes of vibration (flexural vibration) of the MS, which were obtained using Equation (14). As shown in Figure 7, with a MS defect, the amplitude of the excitation frequency in the 2nd and 3rd flexural vibration modes was twice as large as when the defect was absent. In this study, a lowpass filter with a cutoff frequency at 4600 Hz was used to obtain the MCP vibration signal, which scales up to the 3rd mode of flexural vibration. The filtered modes of flexural vibration are FSFB. As shown in Figure 6 and Figure 7, the presence of the MCP’s excitation frequencies, impeller defect’s FSF, and corresponding hydraulic defects are covered in this filtered FSFB. The FSFB is used for the extraction of SF in the next step.

### 4.2. Step 2: Multi-Domain Feature Pool Construction

SF’s are extracted from the FSFB in time, frequency, and TFD. The SF was adopted from a previous study [6]. The representative set of SF’s extracted from the FSFB in the time domain are mean, variance, root amplitude, skewness, root mean square (RMS), clearance factor, impulse factor, kurtosis, shape factor, peak, standard deviation, and crest factor. The time-domain SF’s are presented in Table 1. Continuously, features such as mean frequency, root variance, standard deviation, spectral kurtosis, and root mean frequency are extracted from the FSFB in the frequency domain. Table 2 shows the SF’s extracted from FSFB in the frequency domain. To extract the SF’s from the FSFB in TFD, the FSFB is transformed into TFD using WPT. In this study, Daubechies family db4 mother wavelet is used to decompose the FSFB up to k = 3 levels. As a result, a total of 2 ^k^ WPT bases are obtained. The features mentioned in Table 1 are extracted from each base of WPT. Experimental studies on the selection of optimal wavelets revealed that the Daubechies family db4 mother wavelet is sensitive towards the ongoing processes inside the MCP [46,47]. Therefore, in this study, Daubechies family db4 mother wavelet is selected for decomposition of FSFB. All the extracted SF were combined into a single feature vector called MDFP. The MDFP contains the features of time domain, frequency domain, and TFD. Thus, for each MCP condition, the MDFP contains a total of (12 + 5 + (2 ^k^ × 12) = 113) features.

### 4.3. Step 3: Novel Informative Ratio Principal Component Analysis

The MDFP results in a larger dimension; furthermore, not all features are best for representing the MCP condition and they can affect the accuracy of the condition classification. To address this concern, Ir-PCA was proposed for discriminant feature extraction for MCP fault diagnosis. The steps involved in Ir-PCA are:

Step 1. Calculate the inter-class feature sparseness using Equation (15).

(15) $$I_{i}^{S}=\frac{1}{N}\sum_{n=1}^{N}I_{n,i},$$

Using Equation (16), *I_n,i_* can be obtained as:

(16) $$I_{n,i}=\frac{1}{S_{n}\times (S_{n}-1)}\sum_{j,s=1}^{S_{n}}\left|x_{s,n,i}-x_{j,n,i}\right|,\mathrm{where}\;j,s=1,2,...,S_{n},j\neq s.$$

where *N* represents the classes, *i* is the number of features, *x* represents the feature, *S* is the sample number, and $I_{i}^{S}$ is the inter class sparseness of the features in Equations (15) and (16).

Step 2. Compute the inter-class feature mean $\mu_{n,i}$.

Step 3. Determine the distance between the features of different classes using Equation (17).


(17) $$T_{l}^{d}=\frac{1}{N\times (N-1)}\sum_{p,q=1}^{P}\left|\mu_{q,i}-\mu_{p,i}\right|,p,q=1,2,...,P,p\neq q.$$

Step 4. The informativeness of the feature is calculated using Equation (18).

(18) $$I_{r}=\frac{T_{l}^{d}}{I_{i}^{S}}$$

Step 5. Apply PCA to the features with *I_r_ ≥ 0.5*.

(19) $$\text{Ir-PCA}=PCA(features(I_{r}\geq 0.5)).$$

Using Equation (19), a new set of discriminant features with high between classes distance, reduced within class sparseness, and reduced dimensions were obtained. The new Ir-PCA solves the problem of between class feature distance and within class feature sparseness of traditional PCAs. The new set of features are provided to the K-NN for MCP condition classification where *K* = 3. In this study, K-NN was selected for MCP condition identification because of the low computational cost and simple architecture.

## 5. Results and Performance Evaluation

The dataset used for evaluating the performance of the proposed method consists of 1200 VS. These VS were obtained from the MCP in NC, MSH, MSS, and ID conditions. The MDFP was constructed from the SF extracted from the 1200 VS, where the number of SF in MDTF was *N_mcp_ × V_s_ × I_sf_*. The *N_mcp_* is the classes, VS is the VS instances, and *I_sf_* is the SF extracted from VS in each class. A cross-validation (CV) strategy with n-folds (n = 3) was adopted to validate the proposed method. The dataset was partitioned into n-folds where n-fold were used for classifier testing and the rest of the *n*−1 folds were used for testing the classifier. Out of 1200 samples, 800 were used for classifier training and 400 were used for classifier testing. All samples were selected randomly for each trial. To ensure stability in the classification results, each experiment was performed 20 times.

### Performance Comparison of the Proposed and Reference Methods

From the pre-processing of VS and SF using the proposed method, new fault features were extracted. This study provides a comprehensive evaluation of extracted features for fault diagnosis by comparing it with a TFD features extraction method (WPT-MSVM-PCA) [15], an unsupervised feature pre-processing technique (PCA) [42], and a supervised feature pre-processing method (Tr-LDA) [41]. The comparison matrices used in this study are macro-recall (M_recall_) or true-positive rate (T-PR), macro-precision (M_precision_), the average accuracy of the classification (AAC), and error rate for classification (E_r_). These matrices are calculated using the following equations:

(20) $$T-\mathrm{PR}=\frac{1}{k}\sum_{j=1}^{k}\left(\frac{\left(N_{TP}^{j,m}\right)}{N_{TP}^{j,m}+N_{FN}^{j,m}}\right)\times 100(\%)$$

(21) $$\mathrm{AAC}=\frac{1}{k}\sum_{j=1}^{k}\left(\sum_{m=1}^{L}\frac{N_{TP}^{j,m}}{N_{sanples}}\right)\times 100(\%)$$

(22) $$E_{r}=\frac{1}{k}\sum_{j=1}^{k}\left(\frac{\left(N_{TP}^{j,m})+(N_{FN}^{j,m}\right)}{(N_{TP}^{j,m})+(N_{FN}^{j,m})+N_{TN}^{j,m}+N_{FP}^{j,m}}\right)\times 100(\%)$$

where *k* is the CV folds, true positives $\left(N_{TP}^{j,m}\right)$, true negatives $\left(N_{TN}^{j,m}\right)$, false positives $\left(N_{FP}^{j,m}\right)$, and false negatives $\left(N_{FN}^{j,m}\right)$ identified by the classifier as condition *m*; the iteration of CV folds are *j*, and $N_{samples}^{}$ are the samples in the testing subset as stated in Equations (20)–(22).

**Figure 8:**
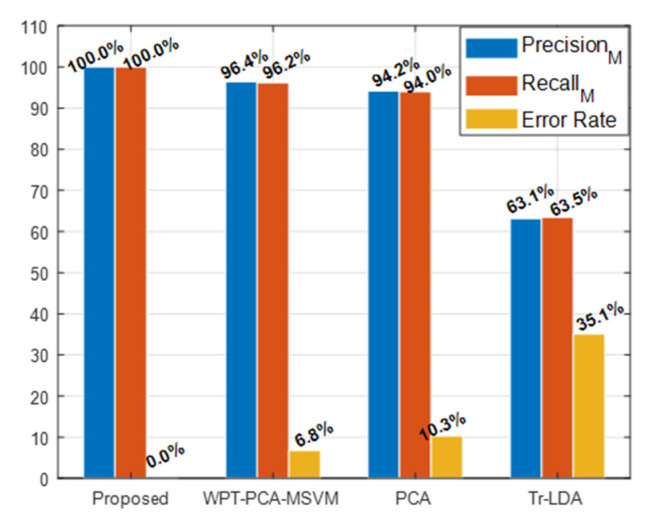
The precision, recall, and errorate obtained from the proposed and reference methods.

**Figure 9:**
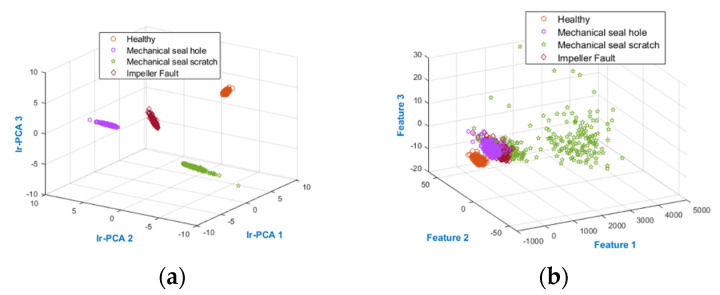

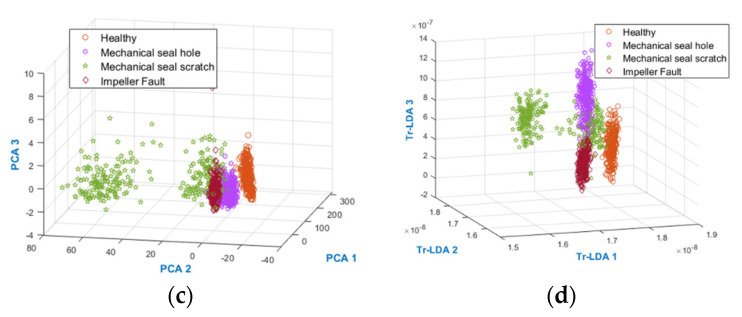
(**a**) Proposed, (**b**) WPT −PCA−MSVM, (**c**) PCA−KNN, and (**d**) Tr−LDA feature spaces.

The results obtained from the proposed and Reference methods are presented in Table 3. It is evident in Table 3 and Figure 8 that the proposed method best identified the MCP working conditions compared with the performance of the reference methods with 0% E_r_, 100% AAC, 100% M_recall_, and 100% M_precision_. The performance of the proposed method is expected because it first calculates the modes of vibration for MCP defects. To overcome the interference macrostructural vibration noise, these MCP defect modes of vibration are filtered from the MCP vibration spectrum. The filtered mode of vibration forms the FSFB, which is then used for multi-domain SF extraction. The multi-domain SF are combined into MDFP, which results in a large dimension. Moreover, some of the features may be noisy and can affect the classifier accuracy. The classifier accuracy is directly proportional to discriminant features. The proposed method applies the novel Ir-PCA to the MDFP to extract discriminant features with reduced dimension. Ir-PCA first assesses the feature informativeness towards the fault by calculating the informative ratio of the features. To obtain a discriminant set of features with reduced dimensions, PCA was applied to the features with a high informative ratio. As shown in Figure 9, the feature space obtained from the proposed method was discriminant; furthermore, the same class features were less sparse. Figure 9 also provides evidence for the higher AAC obtained from the proposed method.

The time–frequency domain (WPT-MSVM-PCA) method pre-processes the vibration signal using WPT and then, with the help of PCA, selects the WPT bases to extract the statistical features. The number of bases defines the dimension of the data. Therefore, the signal is decomposed to 2 levels with 4 bases using WPT. According to PCA, the importance of dynamics connected to data is greater if there are variations in the data, which is why the principal components are sorted by decreasing covariance. Because of the correspondence with 70% data covariance, the first two WPT bases are picked, and the other bases are discarded. After the selection of WPT bases, the useful statistical features are extracted, and then, the best features are picked from the features pool by a wrapper model. Feature extraction and classification using this model produced 6.77% E_r_, 96.39% AAC, 96.39% M_recall_, and 96.20% M_precision_, which are comparatively less than our proposed method, as shown in Table 3 and Figure 8. These results were expected because information loss occurred because of PCA and WPT dependency on signal level decomposition and the use of optimal parent wavelet for WT. Alternatively, the proposed model used all of the bases of WPT, along with the discriminant features obtained from MDFP pre-processing and VS pre-processing. However, sensitivity of WPT-PCA-MSVM to soft defects, such as MSS fault, was observed. The feature space obtained from WPT-PCA-MSVM is shown in Figure 9, where the only separable condition was the NC features. Overall, the AAC of this method was greater than 95% and it can be considered for MCP fault diagnosis.

The unsupervised features processing method PCA is also a dimensionality reduction technique that uses the variance in data to construct data representation in lower dimensions. Application of PCA with a K-NN classifier to our dataset resulted in 10.34% E_r_, 94% AAC, 93.97% M_recall_, and 94.20% M_precision_, which illustrates its poor performance compared with the proposed method, as shown in Table 3 and Figure 8. Additionally, to keep the fault features data, determining the optimal quantity of components is difficult for the PCA. As shown in Figure 9, the classification of features is not as good as the proposed method, resulting in the high classification error value for PCA.

The underperforming supervised features pre-processing method (Tr-LDA) is a linear dimensionality reduction method, which, with the aid of trace ratio criteria, reduces the intraclass dispersion and enhances the interclass distance. The application of this algorithm to our dataset results in 35.09% E_r_, 63.48% AAC, 63.48% M_recall_ and 63.12% M_precision_, which is underperforming compared with our proposed method. This underperformance corroborated expectations because Tr-LDA transforms the data without consideration of feature processing for extracting the intrinsic discriminant information from raw statistical features. Tr-LDA efficiently reduces the intraclass dispersion; however, it results in 35.09% E_r_ in separating different classes, as shown in Figure 9.

Overall, the proposed method considers feature pre-processing and VS pre-processing. The method is easy to implement and efficient for MCP condition classification.

## 6. Conclusions

This paper proposed a new condition classification method for a multistage centrifugal pump. In the signal pre-processing step, the proposed method selected the fault-specific frequency band from the raw vibration signal. The new method for selecting the fault-specific frequency band first calculates the defect vibration modes of the multistage centrifugal pump. Furthermore, to overcome interference macrostructural vibration noise these defect modes are filtered from the raw vibration signal. The filtered modes of vibration formed the fault-specific frequency band. Statistical features in time, frequency, and time–frequency domains were extracted from the fault-specific frequency band. All of the features were combined into a single multi-domain feature pool. To extract discriminant features from a multi-domain feature pool with low dimensions, novel Ir-PCA was applied to the multi-domain feature pool in the feature pre-processing step of the proposed method. The novel Ir-PCA first assesses the feature informativeness towards the fault by calculating the informative ratio of the features. To obtain a discriminant set of features with reduced dimensions, principal component analysis is applied to the features having a high informative ratio. The discriminant set of features obtained from Ir-PCA was then classified using the K-NN classifier for MCP condition classification. The proposed method for multistage centrifugal pump fault diagnosis outperformed the state-of-the-art reference method with an average accuracy of classification of 100%. However, the feature space obtained from the proposed method is highly separable and compact. Therefore, the high variance would be an issue if classification algorithms other than K-NN are used. In the future, the proposed method will be applied to fluid-related defects of the multistage centrifugal pump such as incipient cavitation and severe cavitation.

## Figures and Tables

**Table 1:** Time domain SF’s extracted from the FSFB. The *x*(*n*) is FSFB in the time domain, *N* is the sample data points.

Feature Name	Equation	Feature Name	Equation
Mean	$X_{m}=\frac{\sum_{n=1}^{N}x(n)}{N}$	Variance	$X_{v}=\frac{\sum_{n=1}^{N}{\left(x(n)-X_{m}\right)}^{2}}{\left(N-1\right)}$
Root amplitude	$X_{root}={\left[\frac{\sum_{n=1}^{N}\sqrt{\left|x(n)\right|}}{N}\right]}^{2}$	Skewness	$X_{sk.}=\frac{\sum_{n=1}^{N}{\left(x(n)-X_{m}\right)}^{2}}{(N-1)X_{sd}^{3}}$
RMS	$X_{rms}=\sqrt{\frac{\sum_{n=1}^{N}{\left(x(n)\right)}^{2}}{N}}$	Clearance factor	$X_{clearance}=\frac{X_{peak}}{X_{root}}$
Impulse factor	$X_{impulse}=\frac{X_{peak}}{\frac{1}{N}\sum_{n=1}^{N}\left|x(n)\right|}$	Kurtosis	$X_{kurtosis}=\frac{\sum_{n=1}^{N}{\left(x(n)-X_{m}\right)}^{4}}{(N-1)X_{sd}^{4}}$
Shape factor	$X_{shape}=\frac{X_{rms}}{\frac{1}{N}\sum_{n=1}^{N}\left|x(n)\right|}$	Peak value	$X_{peak}=\max\left|x(n)\right|$
Standard deviation	$X_{sd}=\sqrt{\frac{\sum_{n=1}^{N}{\left(x(n)-X_{m}\right)}^{2}}{N-1}}$	Crest factor	$X_{crest}=\frac{X_{peak}}{X_{rms}}$

**Table 2:** Frequency domain SF’s extracted from the FSFB. The *s*(*k*) is the FSFB spectrum, *k* is the sample of spectrum, and *f_k_* is the value of frequency at spectrum sample *k*.

Feature Name	Equation	Feature Name	Equation
Mean frequency	$X_{mf}=\frac{\sum_{k=1}^{K}s(k)}{K}$	Standard deviation	$\sigma_{{}^{f}}^{2}=\frac{\sum_{k=1}^{K}{\left(s(k)-X_{mf}\right)}^{2}}{K-1}$
Root variance frequency	$Xrvf=\sqrt{\frac{\sum_{k=1}^{K}{\left(s(K)-Xmf\right)}^{2}}{K}}$	Spectral kurtosis	$X_{fkurtosis}=\frac{\sum_{k=1}^{K}{\left(s(k)-X_{mf}\right)}^{4}}{(K)\sigma_{f}^{4}}$
Root mean square frequency	$X_{frms}=\sqrt{\frac{\sum_{k=1}^{K}f_{k}^{2}{\left(s(K)\right)}^{2}}{s(K)}}$		

**Table 3:** Performance of the proposed method and reference methods.

Methods	T-PR (%)				AAC (%)
	NC	MSH	MSSH	ID	
Proposed	100	100	100	100	100
WPT-PCA-MSVM	100	94.11	96.55	94.91	96.39
PCA-KNN	100	92.45	86.45	96.96	94
Tr-LDA	65.42	68.53	52.25	67.74	63.48

## Data Availability

The data presented in this study are available on request from the corresponding author.
